# Predictors of Near-Infrared Spectroscopy-Detected Lipid-Rich Plaques by Optical Coherence Tomography-Defined Morphological Features in Patients With Acute Coronary Syndrome

**DOI:** 10.3389/fcvm.2022.842914

**Published:** 2022-02-21

**Authors:** Eisuke Usui, Taishi Yonetsu, Mari Ohmori, Yoshinori Kanno, Masahiko Nakao, Takayuki Niida, Yuji Matsuda, Junji Matsuda, Tomoyuki Umemoto, Toru Misawa, Masahiro Hada, Masahiro Hoshino, Yoshihisa Kanaji, Tomoyo Sugiyama, Tsunekazu Kakuta, Tetsuo Sasano

**Affiliations:** ^1^Cardiovascular Medicine, Tokyo Medical and Dental University, Tokyo, Japan; ^2^Cardiovascular Medicine, Tsuchiura Kyodo General Hospital, Ibaraki, Japan

**Keywords:** acute coronary syndrome (ACS), cholesterol crystal, lipid-rich plaque, near-infrared spectroscopy (NIRS), optical coherence tomography, thin-cap fibroatheroma (TCFA)

## Abstract

**Background:**

Near-infrared spectroscopy (NIRS) provides the localization of lipid-rich components in coronary plaques. However, morphological features in NIRS-detected lipid-rich plaques (LRP) are unclear.

**Methods:**

A total of 140 *de novo* culprit lesions in 140 patients with the acute coronary syndrome (ACS) who underwent NIRS and optical coherence tomography (OCT) examinations for the culprit lesions at the time of percutaneous coronary interventions were investigated. We defined a NIRS-LRP as a lesion with a maximum lipid core burden index of 4 mm [LCBI_4mm_] > 500 in the culprit plaque. Clinical demographics, angiographic, and OCT findings were compared between the patients with NIRS-LRP (*n* = 54) vs. those without NIRS-LRP (*n* = 86). Uni- and multivariable logistic regression analyses were performed to examine the independent OCT morphological predictors for NIRS-LRP.

**Results:**

Clinical demographics showed no significant differences between the two groups. The angiographic minimum lumen diameter was smaller in the NIRS-LRP group than in the non- NIRS-LRP group. In OCT analysis, the minimum flow area was smaller; lipid angle, lipid length, the prevalence of thin-cap fibroatheroma, and cholesterol crystals were greater in the NIRS-LRP group than in the non-NIRS-LRP group. Plaque rupture and thrombi were more frequent in the NIRS-LRP group, albeit not significant. In a multivariable logistic regression analysis, presence of thin-cap fibroatheroma [odds ratio (OR): 2.56; 95% CI: 1.12 to 5.84; *p* = 0.03] and cholesterol crystals (OR: 2.90; 95% CI: 1.20 to 6.99; *p* = 0.02) were independently predictive of NIRS-LRP.

**Conclusions:**

In ACS culprit lesions, OCT-detected thin-cap fibroatheroma and cholesterol crystals rather than plaque rupture and thrombi were closely associated with a great lipid-core burden.

## Introduction

Near-infrared spectroscopy (NIRS) is a novel modality useful to identify lipid-rich plaques prone to progress (1, 2). NIRS system is combined in conventional intravascular ultrasound (IVUS) imaging catheters and could be used in daily catheterization procedures. Optical coherence tomography is an imaging modality that enables us to identify coronary plaque features with high-resolution imaging quality (3, 4). Several previous studies compared NIRS-IVUS and optical coherence tomography (OCT) findings and showed associations between NIRS-detected great lipid core burden index (LCBI) and OCT-detected plaque vulnerability in stable patients (5) and non-infarct-related arteries (6). Recently, a NIRS-IVUS and OCT study (7) has proposed NIRS-IVUS-derived criteria to predict the OCT-derived plaque morphologies of the culprit lesions of acute myocardial infarction. Nevertheless, OCT-derived predictive variables of NIRS-detected great LCBI are still unknown in culprit lesions of the acute coronary syndrome (ACS) despite great LCBI in ACS culprit lesions has been known to be predictive of periprocedural myocardial injury after stenting (8–12). Thus, this study aimed to examine (i) the relation between OCT-derived plaque morphologies and great LCBI and (ii) the OCT-derived lipid-related predictors of great LCBI in culprit lesions in patients with ACS.

## Methods

### Study Population

This was a multicenter, retrospective, observational study from two hospitals in Japan. In the institutional database of Tokyo Medical and Dental University and Tsuchiura Kyodo General Hospital between July 2015 and October 2021, a total of 506 culprit lesions in 506 patients who underwent both OCT and NIRS examinations before revascularization were investigated. Exclusion criteria were patients with stable coronary artery disease (*n* = 301), in-stent restenosis (*n* = 25), lesions with pre-imaging ballooning or atherectomy (*n* = 32), and poor imaging quality (*n* = 8). In the present study, myocardial infarction was defined as type I or II myocardial infarction in the fourth universal definition (13). In brief, it was defined when there is clinical evidence of acute myocardial ischemia (symptoms of myocardial ischemia; new ischemic electrocardiographic changes; development of pathological Q waves; imaging evidence of new loss of viable myocardium or new regional wall motion abnormality in a pattern consistent with an ischemic etiology; identification of a coronary thrombus by angiography or autopsy) and with detection of a rise and/or fall of cardiac troponin values with at least one value above the 99th percentile URL. ST-segment elevation myocardial infarction was defined as myocardial infarction with ST-segment elevation >0.1 mV in > 2 contiguous leads or a new left bundle-branch block on the electrocardiogram. Non–ST-segment elevation myocardial infarction was defined as myocardial infarction in the absence of ST-segment elevation on the electrocardiogram. Unstable angina was defined as having newly developed/accelerating chest symptoms on exertion or rest angina within 2 weeks without elevated cardiac biomarkers. The culprit lesion was determined by the operator's discretions, employing angiography, electrocardiographic changes, or left ventricular wall motion abnormalities. In patients with multiple stenoses, the culprit lesion was determined as the lesion with the tightest stenosis. Finally, 140 ACS culprit lesions in 140 patients comprised the final dataset. The study complied with the Declaration of Helsinki and was approved by the institutional review boards of Tokyo Medical and Dental University and Tsuchiura Kyodo General Hospital. All the patients provided written informed consent before imaging and subsequent intervention for possible data use in future studies.

### Cardiac Catheterization

Each patient initially underwent standard selective coronary angiography for the assessment of coronary anatomy *via* radial or femoral artery using a 6- or 7-Fr system. Coronary angiograms were analyzed quantitatively using a CMS-MEDIS quantitative coronary angiography (QCA) system (Medis Medical Imaging Systems, Leiden, The Netherlands) to measure the minimum lumen diameter and reference the lumen diameter, percent diameter stenosis, and lesion length at the culprit lesion. All the patients had received a bolus injection of heparin (5,000 IU) before the procedure, and an additional bolus injection of 2,000 IU was administered every hour as needed to maintain an activated clotting time > 250 s. QCA measurements were performed in diastolic frames from orthogonal projections. Post-PCI coronary flow was assessed according to the Thrombolysis in Myocardial Infarction (TIMI) flow grade (14). OCT and NIRS-IVUS imaging were performed before PCI for the culprit lesion.

### OCT Imaging Acquisition and Analysis

OCT was performed after intracoronary nitroglycerin (100–200 μg). Frequency-domain OCT (ILUMIEN OPTIS, Abbott Vascular, Santa Clara, California in 137 patients or Optical Frequency Domain Imaging System, Terumo Corporation, Tokyo, Japan, in three patients) was used. The technique for OCT imaging has been described previously (3, 4). In brief, for frequency-domain OCT systems, a 2.7-Fr (Dragonfly OPTIS or Dragonfly OpStar Abbott Vascular) or a 2.6-Fr (Fastview, Terumo Corporation) catheter was advanced over a guidewire, followed by an automated pullback with a speed of 18–36 mm/s and 180 frames/s (Dragonfly OPTIS and Dragonfly OpStar) or 20–40 mm/s and 160 frames/s (Fastview) with continuous contrast injection (4 ml/s, 14–18 ml total).

**Figure 1:**
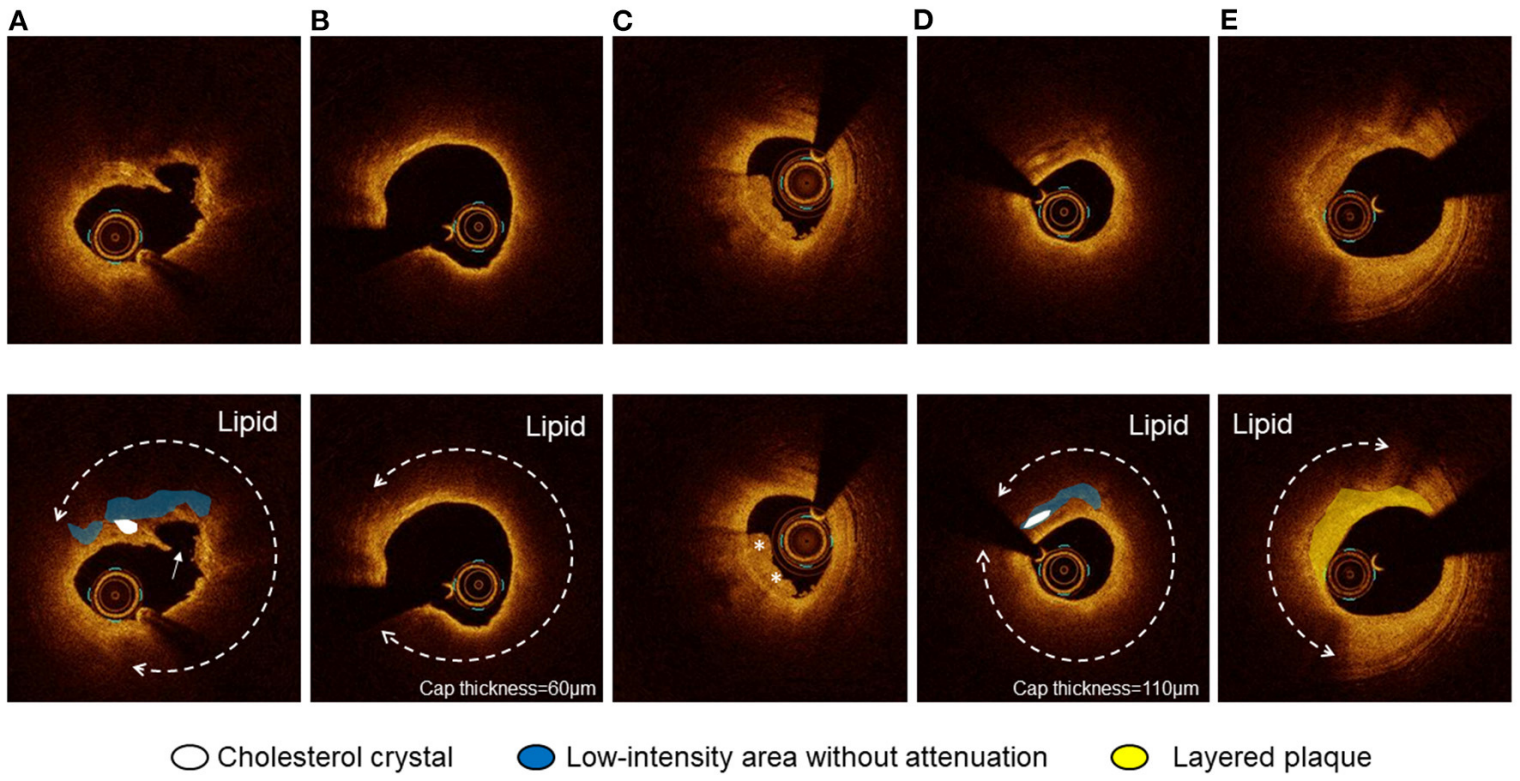
Optical coherence tomography (OCT) images of plaque rupture, thin-cap fibroatheroma (TCFA), thrombus, cholesterol crystal (CC), and layered plaque. **(A)** Plaque rupture with a lipidic plaque and cholesterol crystal accompanying a homogeneous low-intensity area without attenuation. CC was defined as a thin linear region of high intensity, having a clear border with adjacent tissue, not present within or at the border of a calcified plaque. **(B)** TCFA was defined as a lipidic plaque with a lipid angle of > 90° with a fibrous cap thickness <65 μm. **(C)** Thrombus was defined as an irregular mass (diameter > 250 μm) either attached to the luminal surface or floating within the lumen. **(D)** CC accompanying a homogeneous low-intensity area without attenuation. The thinnest fibrous cap thickness was 110 μm. **(E)** The layered plaque was defined as a layer of tissue located close to the luminal surface with clear demarcation from the underlying plaque.

Optical coherence tomography images were analyzed using proprietary software (Abbott or Terumo) by two experienced investigators (EU and TY). A 30-mm segment of the culprit lesion (15-mm proximal and 15-mm distal to the most stenotic lesion site) was examined in all the culprit vessels. Proximal and distal references were defined as the cross-sections with the largest lumen area before major side branches within the examined segment. The reference lumen area was defined as an average of the largest lumen area proximal and distal to the stenosis. The flow area was calculated in each frame as the lumen area minus the thrombus area (15). Percent area stenosis was calculated according to the following formula: [(mean reference lumen area minus minimal lumen area)/mean reference lumen area]. The plaque was categorized as lipidic, fibrous, or calcified. The lipidic plaque had a region with strong signal attenuation and a diffuse border, and a plaque was considered lipid rich if the lipidic angle was > 90°. Plaque rupture was defined as a disrupted fibrous cap with intraplaque cavity formation (Figure 1A). Thin-cap fibroatheroma (TCFA) had a fibrous cap thickness <65 μm with a lipid-rich plaque (Figure 1B) (4). The fibrous plaque had a homogeneous signal-rich region. The calcified plaque was defined as a signal-poor or heterogeneous region with sharply delineated borders. Thrombus was defined as an irregular mass (diameter >250 μm) either attached to the luminal surface or floating within the lumen (Figure 1C) (4). We defined a low-intensity area (LIA) as a homogeneous signal-poor region without attenuation that was ≥0.5 mm in length (Figure 1D) (3, 16, 17). Cholesterol crystal (CC) was defined as a thin linear region of high intensity, having a clear border with adjacent tissue, not present within or at the border of the calcified plaque (Figure 1D) (18). The layered plaque was defined as a layer of tissue located close to the luminal surface with clear demarcation from the underlying plaque (Figure 1E) (19). Macrophages were defined as signal-rich, distinct, or confluent punctate regions accompanied by heterogenic signal shadows. Microvessels were non-signal tubuloluminal structures in a plaque without a connection to the lumen (20).

### NIRS-IVUS Imaging Acquisition

Following the OCT imaging, NIRS-IVUS imaging was performed by using the TVC Imaging System (InfraReDx, Burlington, Massachusetts), including MC8 on a 40-MHz TVC Insight catheter (*n* = 31) or MC10 on a 50-MHz Dualpro catheter (*n* = 109). The NIRS-IVUS catheter was advanced distally to the culprit lesion over a workhorse guidewire, followed by an automated pullback with a speed of 0.5 mm/s for the TVC Insight catheter or 2 mm/s for the Dualpro catheter. The TVC Imaging System provided concurrent color-coded data of a near-infrared spectrum co-registered with IVUS grayscale images. NIRS-IVUS images were stored digitally and analyzed with offline software (echoPlaque 4, INDEC Medical Systems, Inc., Mountain View, California) by an investigator who was blinded to the OCT findings (M.Hada.).

Near-infrared spectroscopy (NIRS) data were denoted on a chemogram, demonstrating the color-coded distribution of lipid in the vessel combined with IVUS images. The chemogram indicated the probability of lipid every 1 pixel (0.1 mm in a longitudinal image and 1° in a circumferential image) by using red (low probability of lipid) and yellow (high probability of lipid). The lipid core burden index (LCBI) was defined as the fraction of pixels, indicating lipid at the probability >0.6 within the region of interest, multiplied by 1,000. The maximum LCBI in 4 mm (maxLCBI_4mm_) was defined as the maximum value of the LCBI for any 4-mm region in the culprit lesion. We defined NIRS-LRP as a lesion having max LCBI_4mm_ of > 500 (8, 10, 11).

### Statistical Analysis

Categorical variables were presented as frequency and were compared with χ^2^ statistics or the Fisher exact test. Continuous variables were presented as median and interquartile range and were compared with the Mann–Whitney *U* test. Prevalence of OCT-derived plaque morphologies in relation to max LCBI_4mm_ on tertiles was compared using Cochran–Armitage trend test with *p* < 0.05 as significant. Multivariable logistic regression analyses were performed to identify the independent predictors of NIRS-LRP. We used a generalized estimating equation approach to compensate for clustering effects of multiple lesions in the same patient. A multivariable model comprised age, gender, and associated variables in the univariate analyses (*p* <0.10) in clinical demographics and OCT characteristics. Although a presence of calcification was positively associated with NIRS-LRP in the univariable analysis, we did not include calcification in the multivariable analysis for the purpose of building a prediction model, which is composed of lipid-related OCT findings. Inter- and intra-observer variability of diagnosis of TCFA and CC was tested by two independent observers and repeated by one observer 4 weeks apart in 50 randomly selected cases using Kappa statistics. A 2-sided *p* < 0.05 was considered statistically significant. Statistical analysis was performed with R statistics version 3.5.3 (R Foundation for Statistical Computing, Vienna, Austria).

## Results

### Baseline Clinical Demographics

Patient age was median 66 (interquartile range; 56.8–73.) years, and 82.9% (116/140) were men. More than half (56.4%) of the patients presented with non-ST-segment elevation myocardial elevation (43 ST-segment elevation MI, 79 non–ST-segment elevation MI, and 18 unstable angina). The prevalence of diabetes mellitus and pre-PCI statin treatment was 30 and 29.3%, respectively. Baseline clinical demographics showed no significant differences between patients with NIRS-LRP and those without NIRS-LRP in culprit lesions (Table 1).

**Table 1:** Clinical demographics, angiographic, and optical coherence tomography findings.

	**Patients with NIRS-LRP (*n* = 54)**	**Patients without NIRS-LRP (*n* = 86)**	***p* value**
**Clinical demographics**
Age, years	67.0 [58.3, 75.0]	65.5 [55.3, 72.0]	0.30
Male	43 (79.6)	73 (84.9)	0.57
Diabetes mellitus	17 (31.5)	25 (29.1)	0.91
Hypertension	36 (66.7)	57 (66.3)	1.00
Dyslipidemia	27 (50.0)	42 (48.8)	1.00
Current smoking	19 (35.2)	37 (43.0)	0.46
Renal insufficiency^*^	19 (35.2)	27 (31.4)	0.78
Prior myocardial infarction	2 (3.7)	6 (7.0)	0.66
Prior percutaneous coronary intervention	0 (0.0)	8 (9.3)	0.053
Clinical presentation			0.11
STEMI	22 (40.7)	21 (24.4)	
Non-STEMI	27 (50.0)	52 (60.5)	
Unstable angina	5 (9.3)	13 (15.1)	
Total cholesterol, mg/dL	195.5 [168.3, 221.8]	192.0 [163.3, 211.8]	0.55
LDL cholesterol, mg/dL	123.0 [103.3, 144.5]	111.0 [90.0, 134.0]	0.053
HDL cholesterol, mg/dL	44.0 [36.0, 54.8]	46.0 [41.0, 55.0]	0.20
Triglycerides, mg/dL	112.0 [69.8, 176.5]	109.0 [81.0, 183.0]	0.34
C-reactive protein, mg/dL	0.14 [0.07, 0.39]	0.16 [0.05, 0.38]	0.99
Pre-admission statin treatment	11 (20.4)	30 (34.9)	0.10
Statin on discharge	52 (96.3)	86 (100.0)	0.29
**Angiographic findings**
Target vessel			0.25
Left anterior descending artery	37 (68.5)	47 (54.7)	
Left circumflex artery	7 (13.0)	10 (11.6)	
Right coronary artery	10 (18.5)	28 (32.6)	
Left main	0 (0.0)	1 (0.1)	
Minimum lumen diameter, mm	0.66 [0.47, 0.85]	0.80 [0.57, 1.00]	0.02
Reference diameter, mm	2.71 [2.32, 3.21]	2.92 [2.40, 3.36]	0.11
Diameter stenosis, %	74.7 [68.9, 83.0]	74.3 [63.3, 78.9]	0.15
Lesion length, mm	12.8 [10.4, 17.1]	12.3 [9.3, 16.1]	0.30
**Optical coherence tomographic findings**
Minimum flow area, mm^2^	1.02 [0.82, 1.15]	1.09 [1.00, 1.42]	<0.01
Area stenosis, %	82.8 [78.9, 87.4]	82.4 [75.1, 87.6]	0.44
Lipidic plaque	53 (98.1)	71 (82.6)	0.01
TCFA	23 (42.6)	16 (18.6)	<0.01
Maximum lipid angle, 0	287.6 [222.5, 360.0]	199.0 [122.9, 296.8]	<0.01
Thinnest fibrous cap, mm	0.07 [0.06, 0.10]	0.07 [0.06, 0.14]	0.54
Lipid length, mm	12.55 [8.40, 17.30]	8.55 [4.62, 12.38]	<0.01
Thrombus	37 (68.5)	53 (61.6)	0.52
Plaque rupture	31 (57.4)	42 (48.8)	0.42
Low-intensity area without attenuation	49 (90.7)	69 (80.2)	0.15
Cholesterol crystal	44 (81.5)	51 (59.3)	0.01
Layered plaque	37 (68.5)	47 (54.7)	0.15
Macrophage accumulation	38 (70.4)	57 (66.3)	0.75
Microvessels	17 (31.5)	35 (40.7)	0.36
Calcification	37 (68.5)	43 (50.0)	0.048
Maximum calcification angle, 0	97.5 [78.0, 130.3]	88.3 [57.0, 136.9]	0.35
Maximum calcification thickness, mm	0.75 [0.53, 1.08]	0.79 [0.59, 1.06]	0.98
Calcified nodule	0 (0)	2 (2)	0.69

*Values are n (%) or median [interquartile range]*.

* *Estimated glomerular filtration rate <60 mL/min/1.73 m^2^ using the Modification of Diet in Renal Disease study equation*.

*HDL, high-density lipoprotein; LDL, low-density lipoprotein; LRP, lipid-rich plaque; NIRS, near-infrared spectroscopy; STEMI, ST-segment elevation myocardial infarction; TCFA, thin-cap fibroatheroma*.

### Angiographic and OCT Findings According to Max LCBI_4mm_

**Figure 2:**
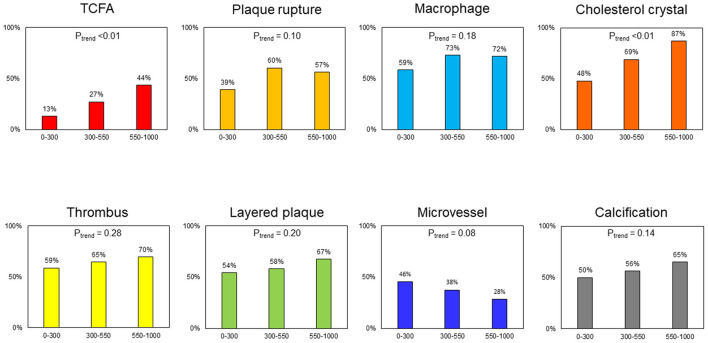
Frequency of plaque morphologies in relation to max lipid core burden index in 4mm (LCBI_4mm_). TCFA and cholesterol crystal tended to be more frequent according to the increase of max LCBI_4mm_ (*P*_trend_ < 0.01). The frequency of plaque rupture, macrophage, thrombus, layered plaque, microvessel, and calcification was similar in relation to max LCBI_4mm_ (*P*_trend_ > 0.05). LCBI_4mm_, lipid core burden index in 4 mm; TCFA, thin-cap fibroatheroma.

Overall, the mean and median values of max LCBI_4mm_were 438 ± 266 and 443 [223–631], respectively. There was good concordance of inter- and intra-observer agreement for the identification of TCFA (κ = 0.77,0.91) and CC (κ = 0.88,0.92). All 95 culprit lesions with OCT-detected CCs also had LIAs in the culprit plaque (Supplementary Figure 1). Table 1 shows the angiographic and OCT findings according to the presence or absence of NIRS-LRP in culprit lesions. The angiographic minimum lumen diameter was smaller in patients with NIRS-LRP than in patients without NIRS-LRP. By OCT, the minimum flow area was smaller; lipidic plaque, TCFA, CC, and calcification were more frequent; maximum lipid angle and lipid length were longer in patients with NIRS-LRP than in patients without NIRS-LRP. The final angiogram showed deteriorated post-PCI TIMI flow grade (Grade 2 or worse) in 3.7% (2/54) of the patients with NIRS-LRP and in 1.2% (1/86) of those without NIRS-LRP (*p* = 0.68). All the other patients showed normal flow grades (Grade 3). The prevalence of thrombus, plaque rupture, low-intensity area without attenuation, layered plaque, macrophage accumulation, and microvessels showed no significant differences between the two groups. The prevalence of OCT-detected TCFA and cholesterol crystals increased according to the increase of max LCBI_4mm_, whereas those of plaque rupture, thrombus, macrophage, layered plaque, microvessel, and calcification were statistically similar irrespective of max LCBI_4mm_ (Figure 2).

Supplementary Figure 2A shows the max LCBI_4mm_ according to the presence or absence of TCFA and cholesterol crystals. Median max LCBI_4mm_ was 608 [428–738], 494 [247–655], 419 [284, 644], and 267 [31–478] in culprit lesions with TCFA and CC, lesions with TCFA without CC, lesions with CC without TCFA, and lesions without TCFA and CC. The absence of both TCFA and CCs showed smaller max LCBI_4mm_ compared with patients having at least 1 characteristic. The presence of CCs was not associated with the prevalence of NIRS-LRP in patients having TCFA, whereas NIRS-LRP was more frequent in patients with CCs under the absence of TCFA (Supplementary Figure 2B). A representative case with NIRS-LRP with a thick fibrous cap is shown in Supplementary Figure 3.

### Predictors of NIRS-LRP

Table 2 shows uni- and multivariable logistic regression analyses to predict the presence of NIRS-LRP in the culprit lesions. TCFA and CC were independently predictive of the presence of NIRS-LRP after adjusting for confounding factors.

**Table 2:** Predictors of NIRS-LRP.

	**OR**	**95% CI**	***p* value**	**OR**	**95% CI**	***p* value**
Age, per 10 years	1.22	0.90–1.63	0.32	1.31	0.93–1.84	0.13
Male	0.70	0.29–1.69	0.42	0.64	0.23–1.84	0.41
STEMI	2.13	1.02–4.43	0.04	1.90	0.83–4.32	0.13
LDL cholesterol, per 10 mg/dL	1.07	0.99–1.17	0.10	–	–	-
Pre-admission statin treatment	0.48	0.22–1.06	0.07	0.47	0.20–1.14	0.09
Thin-cap fibroatheroma	3.25	1.51–6.98	<0.01	2.56	1.12–5.84	0.03
Thrombus	1.36	0.66–2.79	0.41	–	–	-
Plaque rupture	1.41	0.71–2.80	0.32	–	–	-
Low-intensity area without attenuation	2.41	0.84–6.99	0.10	–	–	-
Cholesterol crystal	3.02	1.34–6.79	<0.01	2.90	1.20–6.99	0.02
Layered plaque	1.81	0.88–3.69	0.11	–	–	-
Calcification	2.18	1.07–4.44	0.03	–	–	-

*CI, confidence interval; LDL, low-density lipoprotein; LRP, lipid-rich plaque; NIRS, near-infrared spectroscopy; OR, odds ratio; STEMI, ST-segment-elevation myocardial infarction*.

## Discussion

The major findings of this study are as follows: In the multimodality assessment of the culprit lesions of ACS, (i) prevalence of TCFA and CC increased as max LCBI_4mm_ increased; (ii) prevalence of plaque rupture, thrombus, macrophage, and a layered plaque was similar in any tertiles of max LCBI_4mm_; (iii) TCFA and CC were independently predictive of the presence of NIRS-LRP after a multivariable adjustment.

In the pathological process of coronary atherosclerotic lesion progression, focal macrophage infiltration into an intimal lipid pool triggers loss of matrix and extensive cellular debris with an overlying fibrous cap, leading to the necrotic core formation in the plaque. Intraplaque hemorrhage originated from leaky neoangiogenesis and/or plaque fissure promotes the necrotic core expansion coupled with cholesterol crystallization, followed by fibrous cap thinning and rupture (21, 22). Although OCT provides a high-resolution image and enables us to observe intraplaque structures, the penetration depth is limited and leads to misinterpretation of lipid extent. Thus, we performed a multimodality study to examine OCT-detected plaque features, representing a large amount of lipid using NIRS-detected LCBI as a gold standard.

Jinnouchi et al. reported in an *ex vivo* study (18) that OCT-detected cholesterol crystals were associated with histological stacked cholesterol clefts with high positive and negative predictive values (100 and 84%, respectively) and the presence of intraplaque hemorrhage and late necrotic core, suggesting an ability of OCT-detected cholesterol crystal to identify advanced plaques. Our results that cholesterol crystal was independently associated with large lipid are supported by these pathological data and may help physicians to detect unstable plaques by OCT. Although we tried to detect intraplaque hemorrhage as OCT-detected LIAs (3, 16, 17), this morphology alone did not significantly relate to NIRS-LRP, and 81% (95/118) of them had concomitant CCs. Furthermore, all CCs were concomitant with LIAs (Supplementary Figure 1). LIAs may not always represent intraplaque hemorrhage, and the differential diagnosis can be calcifications and lipid pools in pathological intimal thickening (3). Given that erythrocyte membranes are a principal localized source of free cholesterols in plaques (22, 23), our results suggest that CCs are helpful to identify true intraplaque hemorrhage in OCT-detected LIAs. This explains why we used CCs, not LIA, as one of the vulnerable morphologies in this study.

On the other hand, the prevalence of plaque rupture, thrombus, macrophage, and layered plaque had no significant trend with increasing max LCBI_4mm_. Although plaque rupture should occur based on TCFA with a large amount of lipid, the plaque components could flow out through the rupture, and OCT-detected plaque rupture may sometimes represent a cavity after an old rupture with a small residual lipid. Indeed, 16% (12/73) of OCT-detected plaque ruptures had max LCBI_4mm_ of <200 in this population. This may be the reason why plaque rupture was not associated with NIRS-LRP. Thrombus can be presented not only by plaque rupture due to a large amount of lipid but also by plaque erosion and a calcified nodule (21, 22). Similarly, layered plaques are formed by the healing process after ruptured or erosive thrombotic events (21, 22, 24), which is plausible to be irrelevant with max LCBI_4mm_. In other words, thrombotic events can occur in the absence of a large amount of lipids. Macrophages infiltrate into the lipid pool in pathological intimal thickening and can be observed both in early- and late-stage plaques.

Since TCFA is known to be one of the vulnerable plaque features prone to rupture (21, 22), several studies have tested the effects of intensive medical treatments on fibrous cap thickness (25–27). However, as shown in Supplementary Figure 3, our results suggested that a CC could be a marker of unstable lipid-rich plaques even in the absence of a thin fibrous cap and may help predict distal embolization during PCI procedures (8–12).

### Study Limitations

First, a selection bias cannot be avoided because this was a retrospective observational study at two centers, having a relatively small sample size. Second, the study comprised patients who had undergone both NIRS and OCT examinations before PCI. Thus, we excluded the patients with severe conditions making it hard to undergo these imaging examinations, such as chronic kidney disease, cardiogenic shock, or heavily calcified or tortuous lesions, which may also lead to selection bias. Third, we may not identify intraplaque structures, particularly in the case of lipidic plaques due to the signal attenuation and/or limited penetration depth of OCT. Fourth, OCT findings may not always represent the mechanism of the event because a ruptured plaque may not contain a large amount of lipids after the lipid discharge. Fourth Fifth, the findings can be applied only in culprit lesions in patients with ACS. Fifth Sixth, we need further analyses with clinical outcome data to investigate the prognostic impact of PCI for lesions with NIRS-LRP.

## Conclusion

In ACS culprit lesions, OCT-detected TCFA and CCs rather than plaque rupture and thrombi were closely associated with a great lipid-core burden.

## Data Availability Statement

The original contributions presented in the study are included in the article/Supplementary Material, further inquiries can be directed to the corresponding author.

## Ethics Statement

The studies involving human participants were reviewed and approved by Tsuchiura Kyodo General Hospital, Tokyo Medical and Dental University. The patients/participants provided their written informed consent to participate in this study.

## Author Contributions

EU contributed to resources, data curation, investigation, statistical analysis, and writing. TY contributed to resources, investigation, review, and editing. MHa contributed to resources and investigation. MO, YKann, MN, TN, YM, JM, TU, TM, MHo, TSu, and YKana contributed to resources. TK contributed to resources, review, and editing. TSa contributed to review and editing and supervision. All authors contributed to the article and approved the submitted version.

## Conflict of Interest

The authors declare that the research was conducted in the absence of any commercial or financial relationships that could be construed as a potential conflict of interest.

## Publisher's Note

All claims expressed in this article are solely those of the authors and do not necessarily represent those of their affiliated organizations, or those of the publisher, the editors and the reviewers. Any product that may be evaluated in this article, or claim that may be made by its manufacturer, is not guaranteed or endorsed by the publisher.

## References

[1] Waksman R Di Mario C Torguson R Ali ZA Singh V Skinner WH. Identification of patients and plaques vulnerable to future coronary events with near-infrared spectroscopy intravascular ultrasound imaging: a prospective, cohort study. Lancet. (2019) 394:1629–37. 10.1016/S0140-6736(19)31794-5 31570255

[2] Erlinge D Maehara A Ben-Yehuda O Bøtker HE Maeng M Kjøller-Hansen L. Identification of vulnerable plaques and patients by intracoronary near-infrared spectroscopy and ultrasound (PROSPECT II): a prospective natural history study. Lancet. (2021) 397:985–95. 10.1016/S0140-6736(21)00249-X 33714389

[3] Prati F Regar E Mintz GS Arbustini E Di Mario C Jang IK. Expert review document on methodology, terminology, and clinical applications of optical coherence tomography: Physical principles, methodology of image acquisition, and clinical application for assessment of coronary arteries and atherosclerosis. Eur Heart J. (2010) 31:401–15. 10.1093/eurheartj/ehp433 19892716

[4] Tearney GJ Regar E Akasaka T Adriaenssens T Barlis P Bezerra HG. Consensus standards for acquisition, measurement, and reporting of intravascular optical coherence tomography studies: A report from the International Working Group for Intravascular Optical Coherence Tomography Standardization and Validation. J Am Coll Cardiol. (2012) 59:1058–72. 10.1016/j.jacc.2011.09.079 22421299

[5] Roleder T Kovacic JC Ali Z Sharma R Cristea E Moreno P. Combined NIRS and IVUS imaging detects vulnerable plaque using a single catheter system: A head-to-head comparison with OCT. EuroIntervention. (2014) 10:303–11. 10.4244/EIJV10I3A53 24769522

[6] Zanchin C Ueki Y Losdat S Fahrni G Daemen J Ondracek AS. In vivo relationship between near-infrared spectroscopy-detected lipid-rich plaques and morphological plaque characteristics by optical coherence tomography and intravascular ultrasound: A multimodality intravascular imaging study. Eur Heart J Cardiovasc Imaging. (2021) 22:824–34. 10.1093/ehjci/jez318 31990323

[7] Terada K Kubo T Kameyama T Matsuo Y Ino Y Emori H. NIRS-IVUS for differentiating coronary plaque rupture, erosion, and calcified nodule in acute myocardial infarction. J Am Coll Cardiol Img. (2021) 14:1440–50. 10.1016/j.jcmg.2020.08.030 33221211

[8] Goldstein JA Maini B Dixon SR Brilakis ES Grines CL Rizik DG. Detection of lipid-core plaques by intracoronary near-infrared spectroscopy identifies high risk of periprocedural myocardial infarction. Circ Cardiovasc Interv. (2011) 4:429–37. 10.1161/CIRCINTERVENTIONS.111.963264 21972399

[9] Stone GW Maehara A Muller JE Rizik DG Shunk KA Ben-Yehuda O. Plaque characterization to inform the prediction and prevention of periprocedural myocardial infarction during percutaneous coronary intervention: The CANARY trial (Coronary Assessment by Near-infrared of Atherosclerotic Rupture-prone Yellow). J Am Coll Cardiol Intv. (2015) 8:927–36. 10.1016/j.jcin.2015.01.032 26003018

[10] Kini AS Motoyama S Vengrenyuk Y Feig JE Pena J Baber U. Multimodality intravascular imaging to predict periprocedural myocardial infarction during percutaneous coronary intervention. JAm Coll Cardiol Intv. (2015) 8:937–45. 10.1016/j.jcin.2015.03.016 26088511

[11] Yang HM Yoon MH Lim HS Seo KW Choi BJ Choi SY. Lipid-core plaque assessed by near-infrared spectroscopy and procedure related microvascular injury. Korean Circ J. (2019) 49:1010–8. 10.4070/kcj.2019.0072 31456364 PMC6813158

[12] Matsuoka T Kitahara H Saito K Mori N Tateishi K Fujimoto Y. Utility of near-infrared spectroscopy to detect the extent of lipid core plaque leading to periprocedural myocardial infarction. Catheter Cardiovasc Interv. (2021) 1–10. 10.1002/ccd.29927 34415682

[13] Thygesen K Alpert JS Jaffe AS Chaitman BR Bax JJ Morrow DA. Fourth universal definition of myocardial infarction (2018). J Am Coll Cardiol. (2018) 72:2231–64. 10.1016/j.jacc.2018.08.1038 30153967

[14] TIMI Study Group. The Thrombolysis in myocardial infarction (TIMI) trial. Phase I findings. N Engl J Med. (1985) 312:932–6. 10.1056/NEJM198504043121437 4038784

[15] Xing L Yamamoto E Sugiyama T Jia H Ma L Hu S. EROSION study (Effective Anti-thrombotic therapy without stenting: intravascular optical coherence tomography-based management in plaque erosion): A 1-year follow-up report. Circ Cardiovasc Interv. (2017) 10:1–8. 10.1161/CIRCINTERVENTIONS.117.005860 29246916

[16] Usui E Matsumura M Mintz GS Zhou Z Hada M Yamaguchi M. Clinical outcomes of low-intensity area without attenuation and cholesterol crystals in non-culprit lesions assessed by optical coherence tomography. Atherosclerosis. (2021) 332:41–7. 10.1016/j.atherosclerosis.2021.08.003 34384955

[17] Hoshino M Yonetsu T Yuki Y Inoue K Kanaji Y Usui E. Optical coherence tomographic features of unstable coronary lesions corresponding to histopathological intraplaque hemorrhage evaluated by directional coronary atherectomy specimens. JAm Coll CardiolIntv. (2018) 11:1414–5. 10.1016/j.jcin.2018.04.013 29960754

[18] Jinnouchi H Sato Y Torii S Sakamoto A Cornelissen A Bhoite RR. Detection of cholesterol crystals by optical coherence tomography. EuroIntervention. (2020) 16:395–403. 10.4244/EIJ-D-20-00202 32310132

[19] Shimokado A Matsuo Y Kubo T Nishiguchi T Taruya A Teraguchi I. In vivo optical coherence tomography imaging and histopathology of healed coronary plaques. Atherosclerosis. (2018) 275:35–42. 10.1016/j.atherosclerosis.2018.05.025 29859471

[20] Uemura S Ishigami KI Soeda T Okayama S Sung JH Nakagawa H. Thin-cap fibroatheroma and microchannel findings in optical coherence tomography correlate with subsequent progression of coronary atheromatous plaques. Eur Heart J. (2012) 33:78–85. 10.1093/eurheartj/ehr284 21831910

[21] Virmani R Kolodgie FD Burke AP Farb A Schwartz SM. Lessons from sudden coronary death. Arterioscler Thromb Vasc Biol. (2000) 20:1262–75. 10.1161/01.atv.20.5.1262 10807742

[22] Yahagi K Kolodgie FD Otsuka F Finn A V Davis HR Joner M. Pathophysiology of native coronary, vein graft, and in-stent atherosclerosis. Nat Rev Cardiol. (2016) 13:79–98. 10.1038/nrcardio.2015.164 26503410

[23] Kolodgie FD Gold HK Burke AP Fowler DR Kruth HS Weber DK. Intraplaque hemorrhage and progression of coronary atheroma. N Engl J Med. (2003) 349:2316–25. 10.1056/nejmoa035655 14668457

[24] Burke AP Kolodgie FD Farb A Weber DK Malcom GT Smialek J. Healed plaque ruptures and sudden coronary death: Evidence that subclinical rupture has a role in plaque progression. Circulation. (2001) 103:934–40. 10.1161/01.CIR.103.7.934 11181466

[25] Hattori K Ozaki Y Ismail TF Okumura M Naruse H Kan S. Impact of statin therapy on plaque characteristics as assessed by serial OCT, grayscale and integrated backscatter-IVUS. JAm Coll Cardiol Img. (2012) 5:169–77. 10.1016/j.jcmg.2011.11.012 22340823

[26] Komukai K Kubo T Kitabata H Matsuo Y Ozaki Y Takarada S. Effect of atorvastatin therapy on fibrous cap thickness in coronary atherosclerotic plaque as assessed by optical coherence tomography: The EASY-FIT study. J Am Coll Cardiol. (2014) 64:2207–17. 10.1016/j.jacc.2014.08.045 25456755

[27] Yano H Horinaka S Ishimitsu T. Effect of evolocumab therapy on coronary fibrous cap thickness assessed by optical coherence tomography in patients with acute coronary syndrome. J Cardiol. (2020) 75:289–95. 10.1016/j.jjcc.2019.08.002 31495548

