# Supplementary material for: Predictors of Near-Infrared Spectroscopy-Detected Lipid-Rich Plaques by Optical Coherence Tomography-Defined Morphological Features in Patients With Acute Coronary Syndrome
Source: Front Cardiovasc Med. 2022 Feb 21;9:842914. doi: 10.3389/fcvm.2022.842914 (PMC8899395; doi:10.3389/fcvm.2022.842914)
Supplement: Supplementary file 1 [file Data_Sheet_1.docx]

Supplementary Material

## Supplementary Figures


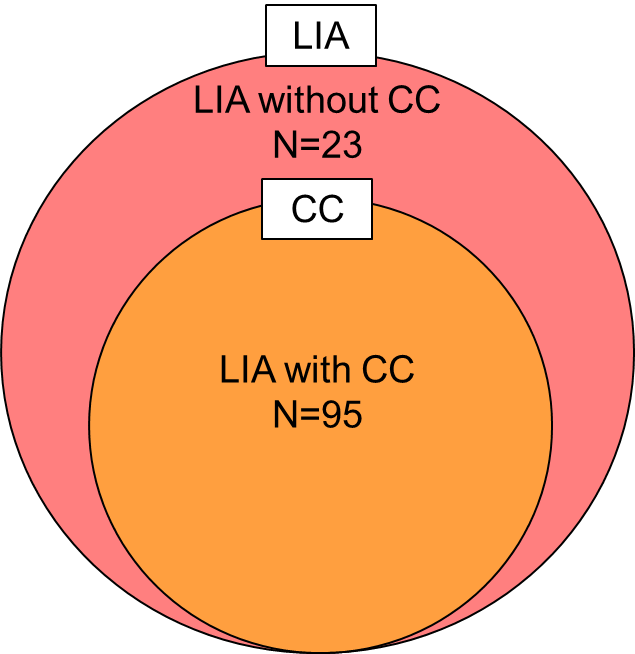


**Supplementary Figure 1.** **Association Between Low-Intensity Area Without Attenuation and Cholesterol Crystals**

All 95 culprit lesions with cholesterol crystals also had low-intensity areas without attenuation in the culprit plaque. CC = cholesterol crystal; LIA = low-intensity area without attenuation.


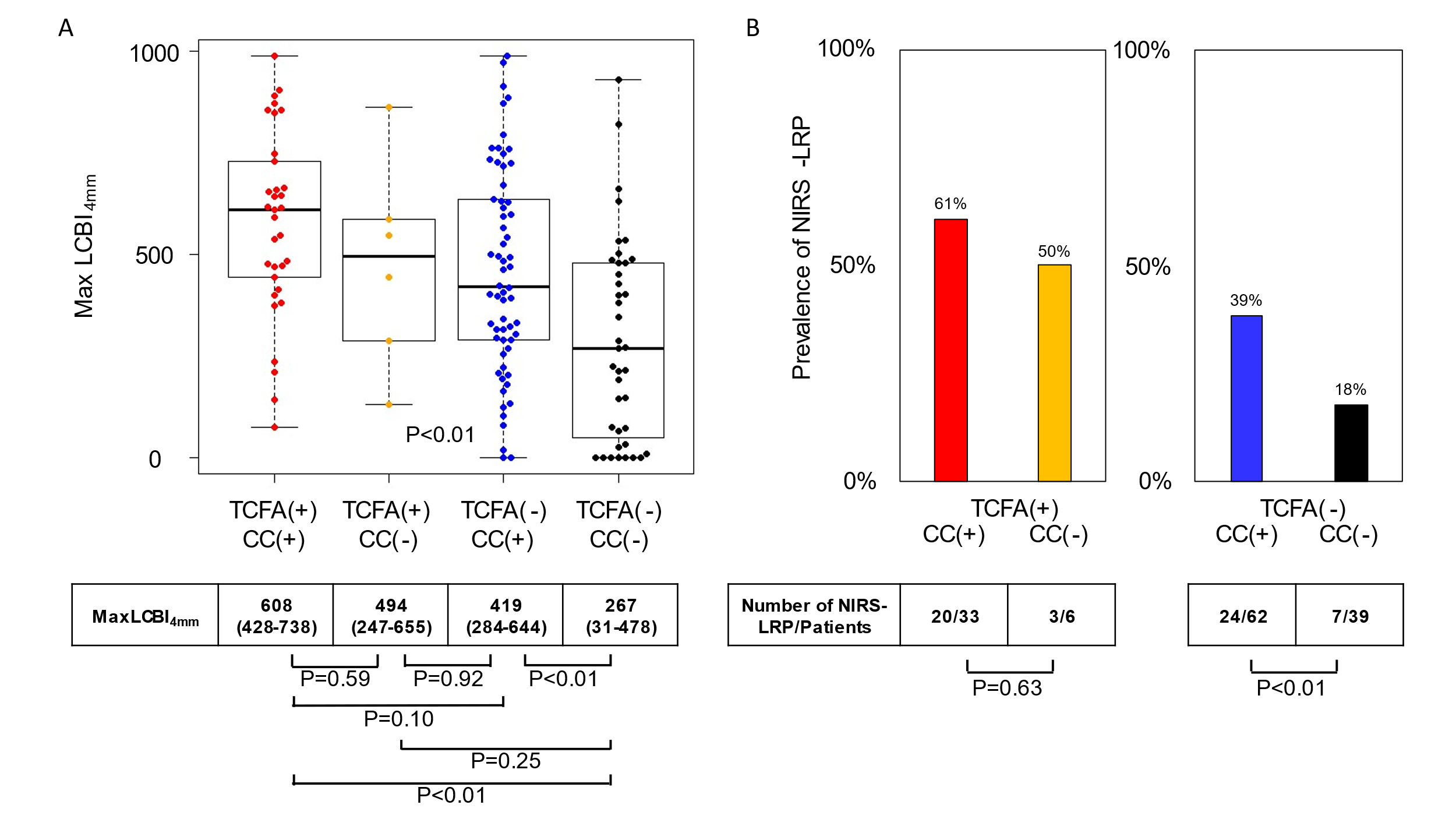
**Supplementary Figure 2.** **Lipid Extent in Relation to Presence or Absence of TCFA and Cholesterol Crystals**

(A) Median max LCBI_4mm_ was 608 [428-738], 494 [247-655], 419 [284, 644], and 267 [31-478] in culprit lesions with TCFA and cholesterol crystal (CC), lesions with TCFA without CC, lesions with CC without TCFA, and lesions without TCFA and CC. (B) Prevalence of NIRS-LRP with or without TCFA. In the absence of TCFA, CC was associated with frequent NIRS-LRP. The absence of both TCFA and CC precluded the presence of NIRS-LRP with a probability of 82%. CC = cholesterol crystal; LRP = lipid-rich plaque; NIRS = near-infrared spectroscopy; TCFA = thin-cap fibroatheroma.


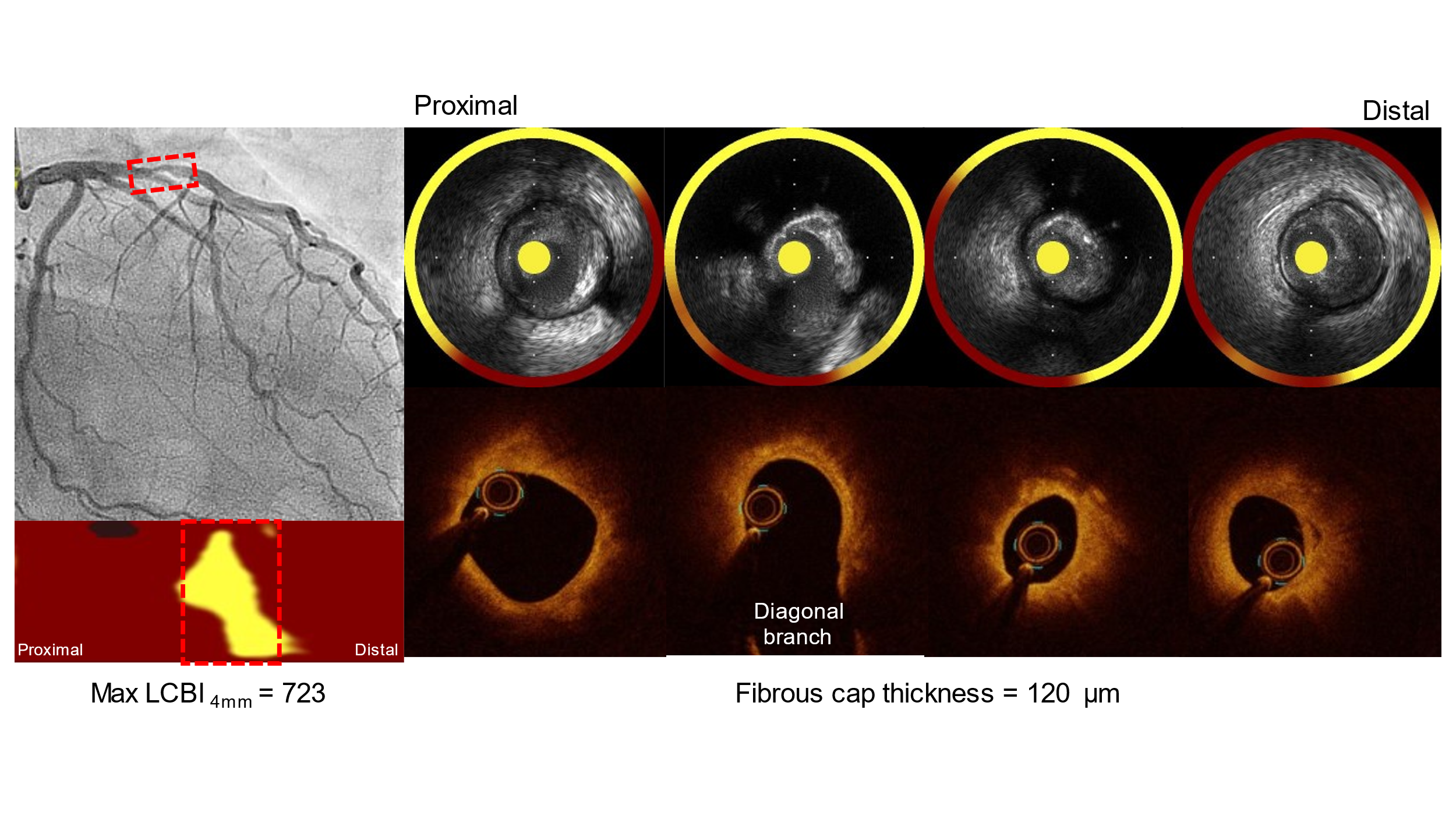


**Supplemental Figure 3. A Representative case of a NIRS-LRP Without OCT-Detected TCFA**

A representative case with a NIRS-LRP without OCT-detected TCFA. A 69-year-old man presented with non-ST-elevation myocardial infarction. Urgent coronary angiography showed a tight stenosis at the proximal left anterior descending artery with max LCBI_4mm_ = 723 by NIRS-IVUS. OCT showed no TCFA and rupture, but cholesterol crystals were present in the culprit lesion. The thinnest fibrous cap thickness was 120μm. IVUS = intravascular ultrasound; LCBI_4mm_ = lipid core burden index in 4mm; LRP = lipid-rich plaque; NIRS = near-infrared spectroscopy; OCT = optical coherence tomography; TCFA = thin-cap fibroatheroma.
